# Public health round-up

**DOI:** 10.2471/BLT.16.011216

**Published:** 2016-12-01

**HIV self-testing in southern Africa**

A****community distributor (right) of HIV tests in Zimbabwe explains how self-testing works.

**Figure:**
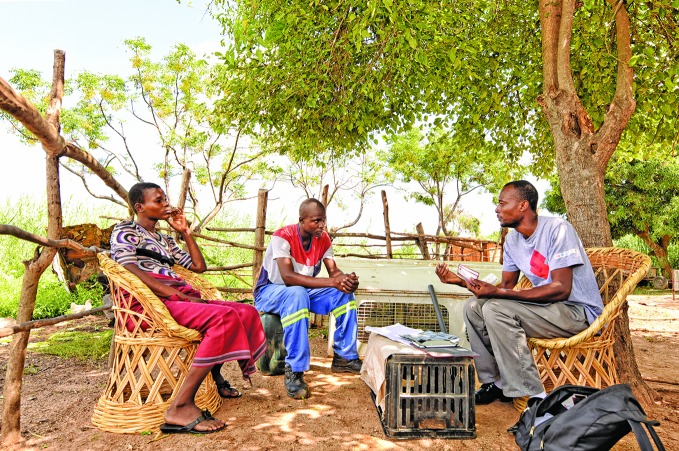
UNITAID/Eric Gauss

## HIV self-testing

For the first time the World Health Organization (WHO) is recommending that individuals should be encouraged to test themselves for HIV infection with quality-assured simple-to-use rapid diagnostic tests.

WHO is also recommending so-called assisted partner notification, which means that anyone whose test result is confirmed positive should contact their sexual partners to let them know with the help of a trained professional.

According to a new WHO guideline released on World AIDS Day on 1 December, HIV self-testing should be considered as an additional approach to conventional HIV testing services.

Guidelines on HIV self-testing and partner notification are intended as a supplement to WHO’s consolidated guidelines on HIV testing services.

“Programmes should evaluate their existing HIV testing approaches and determine where and how to implement HIV self-testing so that it is complementary and addresses gaps in current coverage,” a policy brief on the guidance says.

HIV self-testing is when an individual collects his or her own saliva or blood sample, performs an HIV test and interprets the result alone or with a trusted person. The diagnostic tests may be distributed by trained providers or peers or bought over the counter in pharmacies or through the Internet.

For decades health workers and other people have informally tested themselves for HIV, but it is the first time that WHO has recommended self-testing.

As with all HIV testing, the result of a single rapid diagnostic test is not sufficient to diagnose HIV infection.

According to the new guidance, self-testers who produce a reactive test result (HIV positive) are advised to receive further testing from a trained tester using a validated national algorithm to confirm the result and provide a diagnosis. This should be linked to an assessment for HIV treatment.

## Safe, positive pregnancy

Pregnant women should have at least eight antenatal contacts with health workers – four were previously recommended – and should start seeking care as soon as they know they are pregnant, according to a new WHO guideline.

These are some of the 49 new recommendations to improve the quality of antenatal care and reduce the risk of stillbirths and pregnancy complications.

Last year, an estimated 303 000 women died from pregnancy-related causes, 2.7 million babies died during the first 28 days of life and 2.6 million babies were stillborn. Quality health care during pregnancy and childbirth can prevent many of these deaths.

The guideline, entitled *WHO recommendations on antenatal care for a positive pregnancy experience*, was prepared by the departments of Reproductive Health and Research, Nutrition for Health and Development and Maternal, Newborn, Child and Adolescent Health at WHO headquarters.

It is intended for use by policy-makers, programme managers, nongovernmental and other organizations and health professionals in providing antenatal health care when designing and planning maternal and child care and aims to provide pregnant women with respectful, individualized, person-centred care at every contact.

www.who.int/reproductivehealth/publications/maternal_perinatal_health/anc-positive-pregnancy-experience

## Rapid response to Mosul

WHO has set up 82 rapid response teams to provide health services and prevent disease epidemics among displaced people from Mosul, Iraq’s second city.

In addition, 18 mobile medical clinics and nine ambulances have been deployed in and around Mosul to provide life-saving care and medicines for the displaced.

According to contingency plans, WHO and the rest of the humanitarian community estimate that up to 700 000 people might flee the city and its surrounding areas. Of these, more than 200 000 people will require emergency health services.

Children living in Mosul have reportedly not been systematically immunized since June 2014, when humanitarian access into the city was last possible.

As increasing numbers of people flee Mosul, water and sanitation services in hosting camps may come under severe strain, increasing the risk of waterborne diseases such as cholera. Foodborne and vector-borne diseases also pose a risk.

Eleven of the 82 teams are working on detection and response to outbreaks; 39 are ready to treat civilians exposed to chemical agents; and 32 are responsible for immunization.

The teams are based in the nearby governorates of Ninewa, Kirkuk, Dahuk and Erbil. Trained and supported by WHO, these teams will be sent by the local health authorities to screening sites, camps and other venues in Iraq that are hosting the displaced.

The response teams were established with the support of the European Union and the Office of US Foreign Disaster Assistance, but more funding is needed. As part of the Mosul Flash appeal for preparedness, US$ 35 million has been requested.

http://www.emro.who.int/irq/iraq-news/who-establishes-rapid-response-teams-to-safeguard-the-health-of-newly-displaced-people-from-mosul-iraq.html

**Cover photo**

This month’s cover photo shows an Iraqi family displaced by fighting in the village of Shora walking towards an Iraqi army checkpoint in Mosul district in Iraq.

**Figure:**
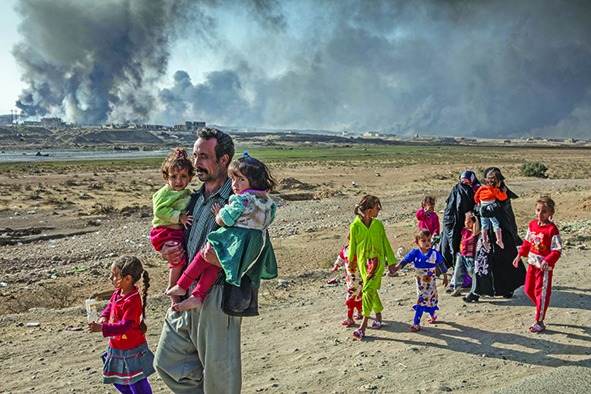
© UNHCR/Ivor Prickett

## Stop surgical infections

WHO issued a list of 29 recommendations last month on how to prevent infections during surgery and slow the development of antibiotic resistance.

Surgical infections are caused by pathogens that enter the body through incisions made during surgery. These infections threaten the lives of millions of patients every year and contribute to the spread of drug-resistant pathogens.

*Global guidelines for the prevention of surgical site infection* is the first set of internationally applicable and evidence-based guidelines on preventing surgical infections and complement WHO’s *Surgical safety checklist* released in 2008.

The guidelines include 13 recommendations for the period before surgery and 16 for preventing infections during and after surgery. They range from simple precautions such as ensuring that patients bathe or shower before surgery and the best way for surgical teams to clean their hands, to advice on when to use antibiotics to prevent infections.

According to WHO estimates, surgical site infections are the most prevalent health care-associated infection in low- and middle-income countries, affecting 11% of surgical patients. 

The incidence of surgical site infections is much lower in high-income countries, but it remains the leading cause of health-care associated infections in Europe and the United States of America (USA).

The economic cost of surgical infections can also be high. In the USA, surgical infections contribute to patients spending more than 400 000 extra days in hospital at a cost of up to US$ 10 billion per year.

http://www.who.int/gpsc/ssi-guidelines

## Marketing unhealthy food

Countries need to control the digital marketing of unhealthy food and non-alcoholic drinks that is directed at children more effectively to help reduce obesity, according to a new report by WHO’s Regional Office for Europe.

“Children across Europe access digital media avidly, predominantly on mobile devices, generally favouring social media and video viewing sites,” the report notes.

One of the key factors for childhood obesity is the marketing of unhealthy foods and drinks to children and a key recommendation of the WHO Commission in Ending Childhood Obesity is to reduce children’s exposure to such marketing.

As a result, WHO has called on its Member States to act. The new report, entitled *Tackling food marketing to children in a digital world: trans-disciplinary perspectives*, advises governments on how they can restrict such marketing in the digital sphere.

The report calls on governments to acknowledge their duty to protect children from digital marketing of products that are high in saturated fat, salt and free sugars and to extend any existing restrictions on conventional marketing of such products to online marketing.

The report encourages governments to draw on existing legislation, regulation and regulatory agencies when developing strategies to protect children from such marketing.

http://www.euro.who.int/en/2016/digital-marketing-to-children

## Extending treatment 

A new tuberculosis drug with a novel mechanism of action, delamanid, which initially was recommended only for adults, is now recommended for the treatment of children with multidrug-resistant tuberculosis (MDR-TB) from the age of six years, according to a new WHO guideline.

An estimated 580 000 people acquired MDR–TB in 2015, with children accounting for about 30 000 of them.

MDR–TB is a form of tuberculosis that is resistant to at least isoniazid and rifampicin, the two most powerful anti-tuberculosis drugs. Patients with this form of tuberculosis can be treated and cured with second-line drugs, but these are more toxic, far more expensive, and require longer treatment duration.

WHO recommended the use of delamanid for the treatment of MDR–TB in adults in 2014, but not for children due to the lack of evidence at the time.

Since then, new data have emerged on the use of delamanid in children and adolescents with MDR–TB. WHO convened an international panel of independent and multidisciplinary experts to assess the new evidence and develop recommendations for the treatment of paediatric MDR–TB.

Based on the panel’s assessment, WHO recommends that delamanid may be added to the WHO-recommended longer regimen in children and adolescents aged 6–17 years.

*The use of delamanid in the treatment of multidrug-resistant tuberculosis in children and adolescents: Interim policy guidance* was released in October.

http://www.who.int/tb/publications/Delamanid_interim_policy

**Looking ahead**

**23 January–1 February 2017 – 140th WHO Executive Board meeting**

4 February 2017 – World Cancer Day

